# Sildenafil-associated severe cholestatic drug-induced liver injury presenting as persistent jaundice after stone extraction

**DOI:** 10.1007/s12328-026-02390-5

**Published:** 2026-07-20

**Authors:** Yuma Kamijo, Takanobu Iwadare, Ryoko Endo, Minori Kamata, Kaede Fujita, Shunichi Wakabayashi, Hiroyuki Kobayashi, Tomoo Yamazaki, Takeshi Uehara, Takefumi Kimura

**Affiliations:** 1 Department of Medicine, Division of Gastroenterology, Shinshu University School of Medicine, 3-1-1 Asahi, Matsumoto, Nagano, 390-8621 Japan; 2 Shinshu University Hospital, Matsumoto, Japan; 3 Department of Laboratory Medicine, Shinshu University School of Medicine, Matsumoto, Japan; 4 Consultation Center for Liver Diseases, Shinshu University Hospital, Matsumoto, Japan; 5 Institute for Biomedical Sciences, Research Cluster for Social Implementation, Matsumoto, Japan

**Keywords:** Sildenafil, Drug-induced liver injury, Cholestatic liver injury, RECAM-J 2023, KAMAGRA®

## Abstract

**Background:**

Sildenafil is widely used as a phosphodiesterase type 5 inhibitor for erectile dysfunction. Drug-induced liver injury (DILI) associated with sildenafil is rare, with severe cholestatic cases remaining poorly characterized.

**Case Summary:**

A 43-year-old Japanese man was assigned to work in the Philippines two months before presentation and began regularly taking sildenafil (KAMAGRA®) obtained locally. One month before presentation, he developed abdominal pain and vomiting. Magnetic resonance imaging revealed choledocholithiasis, and endoscopic retrograde cholangiopancreatography with stone extraction was successfully performed. However, his severe jaundice persisted and progressively worsened. Upon returning to Japan for further evaluation, imaging studies showed no evidence of persistent biliary obstruction**.** Liver biopsy demonstrated marked canalicular cholestasis without bile duct destruction, which was inconsistent with prolonged obstructive jaundice. Causality assessment using revised electronic version of Roussel Uclaf Causality Assessment Method for the diagnosis of drug-induced liver injury-Japan, 2023 (RECAM-J 2023) yielded a score of 11 for sildenafil, indicating a highly probable association. After treatment with ursodeoxycholic acid and colestimide, his bilirubin levels gradually normalized.

**Conclusion:**

Sildenafil formulations obtained outside routine medical supervision may cause severe cholestatic DILI. Careful medication history taking and structured causality assessment are essential for an accurate diagnosis.

## Introduction

Phosphodiesterase type 5 inhibitors act primarily on the corpus cavernosum and pulmonary vasculature, being widely used for the treatment of erectile dysfunction and pulmonary hypertension [1, 2]. Sildenafil is generally regarded as safe and well tolerated; however, rare cases of drug-induced liver injury (DILI) have been reported [3–8]. In particular, the clinical features and outcomes of severe cholestatic liver injury from sildenafil remain insufficiently characterized.

As DILI is largely a diagnosis of exclusion, establishing causality in clinical practice can be challenging. Several structured assessment tools have been developed to improve diagnostic accuracy. The revised electronic version of Roussel Uclaf Causality Assessment Method for the diagnosis of DILI–Japan 2023 (RECAM-J 2023) [9] is a Japanese adaptation of the RECAM system originally proposed in 2022 that incorporates modifications reflecting Japanese clinical practice. This scoring system has recently been introduced as a useful method for assessing suspected DILI. We herein describe a case of prolonged severe cholestatic liver injury associated with sildenafil obtained abroad, in which causality was supported by the RECAM-J 2023 assessment system.

### Case report

The patient was a 43-year-old Japanese man who presented to our hospital with jaundice. His medical history included hypertension and dyslipidemia, for which he had been taking amlodipine 5 mg and rosuvastatin 2.5 mg, respectively, once daily for two years. He reported no recent use of dietary supplements, significant alcohol consumption, or family history of liver disease. No history of blood transfusion, consumption of raw or undercooked meat or wild game, or high-risk sexual exposure associated with viral hepatitis were noted.

Two months before presentation, the patient was assigned to work at a factory in the Philippines. Around the same time, he started sexual relations with a local partner approximately once weekly and consistently used sildenafil 100 mg (KAMAGRA®) obtained from a local acquaintance. One month before presentation, he began experiencing abdominal pain and vomiting, which prompted a visit to a local medical facility. Sildenafil had been continued until shortly before his visit to the local hospital and was discontinued thereafter. Blood tests revealed elevated hepatobiliary enzymes (aspartate aminotransferase [AST]: 157 U/L, alanine aminotransferase [ALT]: 381 U/L, alkaline phosphatase [ALP]: 186 U/L) and jaundice (total bilirubin: 7.2 mg/dL). Magnetic resonance imaging (MRI) demonstrated common bile duct stones with bile duct dilatation. A diagnosis of obstructive jaundice secondary to choledocholithiasis was made, and endoscopic retrograde cholangiopancreatography with stone extraction was performed.

**Fig. 1:**
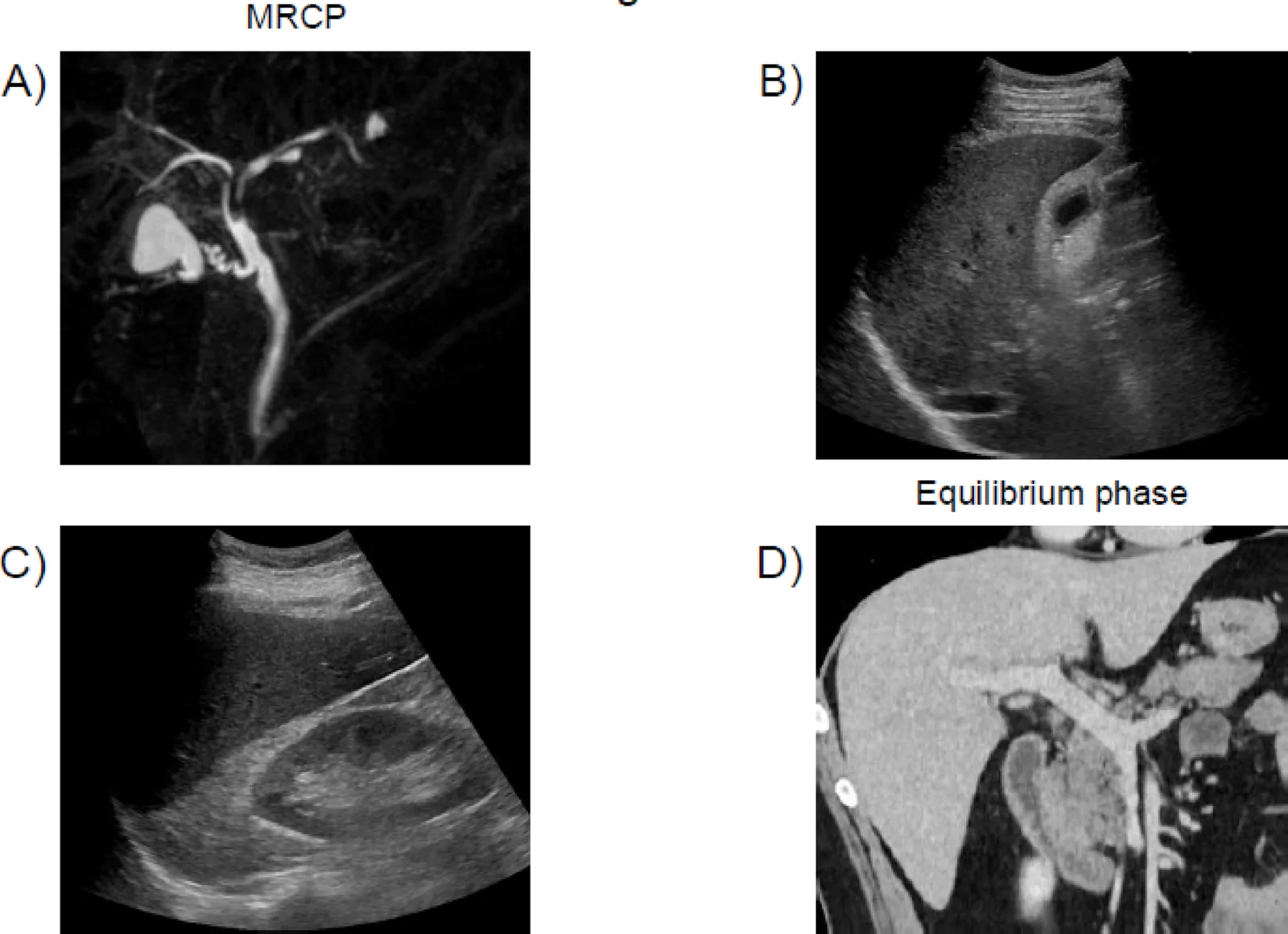
Abdominal imaging findings. **A** Magnetic resonance cholangiopancreatography revealed no evidence of biliary obstruction after endoscopic stone extraction. **B**, **C** Abdominal ultrasonography showed an atrophic gallbladder with mild wall thickening. Hepatic echogenicity was comparable to that of the renal cortex, and the liver surface was smooth. **D** Contrast-enhanced abdominal computed tomography demonstrated mild hepatomegaly without evidence of portal vein thrombosis

Although the patient’s hepatobiliary enzyme levels gradually improved after the procedure, his total bilirubin remained markedly elevated and further increased to 28 mg/dL. As continuing work became difficult, the patient returned to Japan. He subsequently consulted an occupational physician and was treated with ursodeoxycholic acid 600 mg/day, L-carnitine 3000 mg/day, and antihistamines 120 mg/day on an outpatient basis; however, his hyperbilirubinemia persisted. Follow-up MRI revealed no evidence of biliary obstruction (Fig. 1A). He was subsequently referred to our hospital for further evaluation and admitted.

Physical examination disclosed a height of 181.0 cm, weight of 102.0 kg, and body mass index of 31.1 kg/m^2^. The sclerae were icteric, and marked jaundice of the trunk was observed. The abdomen was flat and soft, without tenderness. Mild asterixis was noted. Laboratory tests revealed mildly prolonged prothrombin time (61%), elevated total bilirubin (31.4 mg/dL) and direct bilirubin (21.5 mg/dL), as well as decreased serum albumin (2.8 g/dL), which corresponded to a MELD score 18 points (Table 1). His AST was 54 U/L, ALT was 41 U/L, gamma-glutamyl transferase was 37 U/L, and ALP was 212 U/L, indicating only mild elevation of transaminases and ALP. Serum ammonia was within normal range. Fibrosis markers were elevated for hyaluronic acid (176.5 ng/mL), mac-2 binding protein glycosylation isomer (6.16 COI), autotaxin (1.497 mg/L), and type IV collagen 7S (28.1 ng/mL).

**Table 1:** Laboratory data on admission

*Hematology*			D-Bil	21.5	mg/dL	*Infection markers*		
White blood cells	9.7	× 10^3^/μL	UA	2.9	mg/dL	HBs-Ag	(-)	
Red blood cells	382	× 10^4^/μL	BUN	11	mg/dL	HBs-Ab	(-)	
Hemoglobin	11.8	g/dL	Cr	1.13	mg/dL	HBc-Ab	(-)	
Hematocrit	32.5	%	eGFR	58	mL/min/1.73 m^2^	HCV-Ab	(-)	
MCV	85.1	fL	Na	137	mmol/L	IgM-HA-Ab	(-)	
MCHC	36.3	g/dL	K	3.4	mmol/L	HEV-IgA-Ab	(-)	
Platelet count	45.4	× 10^4^/μL	Cl	103	mmol/L	*Hormones*		
*Coagulation*			Ca	9.1	mg/dL	fT3	2.00	pg/mL
PT%	61	%	NH3	50	μg/dL	fT4	1.15	ng/dL
PT-INR	1.37		LDH	285	U/L	TSH	0.92	μIU/mL
APTT	29.7	sec	*Immunological/serological*		*Virus markers*			
*Chemistry*			CRP	0.60	mg/dL	HSV-IgM	(-)	
Albumin	2.8	g/dL	IgM	44	mg/dL	HSV-IgG	(-)	
Total protein	6.4	g/dL	IgG	1118	mg/dL	CMV-IgM	(-)	
AST	54	U/L	ANA	< 40		CMV-IgG	194	AU/mL
ALT	41	U/L	AMA-M2	< 1.5		EB-VCA-IgM	(-)	
ALP	212	U/L	*Tumor markers*			EB-VCA-IgG	40	
GGT	37	U/L	AFP	1.8	ng/mL	EB-EBNA	40	
T-Bil	31.4	mg/dL	PIVKA-II	3670	mAU/mL	HIV Ag/Ab	0.2	

AFP, Alpha-fetoprotein: ALP, Alkaline phosphatase: ALT, Alanine aminotransferase: AMA-M2, Anti-mitochondrial M2 antibody: ANA, Antinuclear antibody: APTT, Activated partial thromboplastin time: AST, Aspartate aminotransferase: BUN, Blood urea nitrogen: Ca, Calcium: Cl, Chloride: CMV, Cytomegalovirus: CRP, C-reactive protein: Cr, Creatinine: D-Bil, Direct bilirubin: EB-VCA, Epstein-Barr Viral Capsid Antigen: EBNA, EBV Nuclear Antigen: eGFR, Estimated glomerular filtration rate: fT3, Free T3: fT4, Free T4: GGT, Gamma-glutamyltransferase: HA, Hyaluronic acid: HBc-Ab, Hepatitis B core antibody: HBs-Ab, Hepatitis B surface antibody: HBs-Ag, Hepatitis B surface antigen: HCV-Ab, Hepatitis C virus antibody: HEV-IgA, Hepatitis E Virus-Immunoglobulin A: HIV, Human Immunodeficiency Virus: HSV, Herpes Simplex Virus: IgG, Immunoglobulin G: IgM, Immunoglobulin M: K, Potassium: LDH, Lactate dehydrogenase: MCHC, Mean Corpuscular Hemoglobin Concentration: MCV, Mean Corpuscular Volume: Na, Sodium: NH3, Ammonia: P, Phosphate: PIVKA-II, Protein induced by vitamin K absence-II: PT%, Prothrombin time percentage: PT-INR, Prothrombin time—international normalized ratio: T-Bil, Total bilirubin: TSH, Thyroid-stimulating hormone: UA, Uric acid

**Fig. 2:**
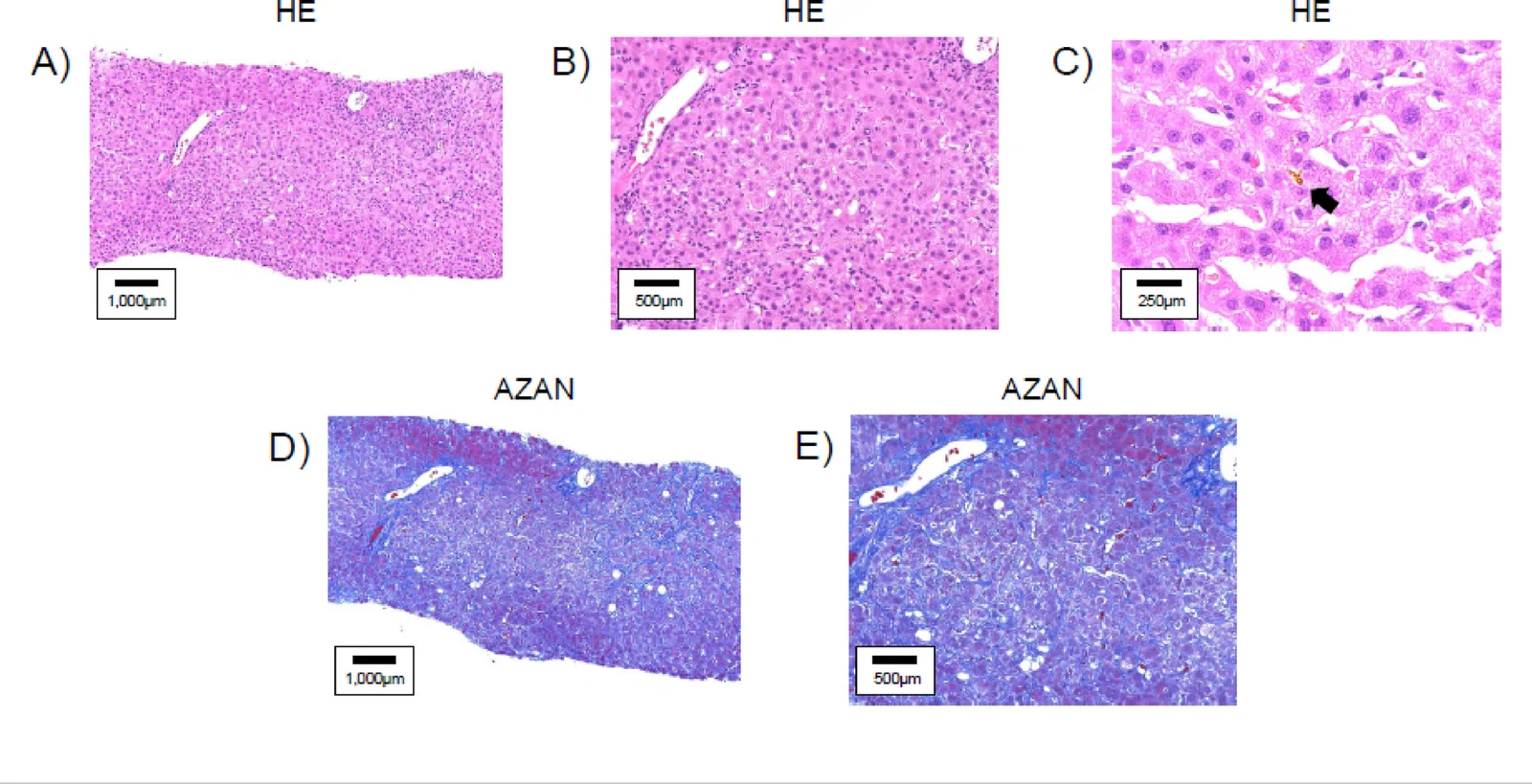
Histopathological liver biopsy findings. **A**, **B**, **C** Hematoxylin and eosin staining showed mild inflammatory cell infiltration in the portal tracts that was predominantly composed of lymphocytes. Prominent canalicular and lobular cholestasis (arrow) were observed. No bile duct destruction, cholangitis, or interface hepatitis were detectable (D, E) Azan staining demonstrated no significant hepatic fibrosis.

Serological testing was negative for hepatitis B virus, hepatitis C virus, and other viral infections, as well as for autoimmune liver diseases (antinuclear antibody: < 40, anti-mitochondrial M2 antibody: < 1.5). Drug-induced lymphocyte stimulation tests for sildenafil and rosuvastatin were negative. Abdominal ultrasonography revealed a heterogeneous hepatic architecture, hepatomegaly (liver anteroposterior diameter: 179.2 mm), gallbladder atrophy, and splenomegaly　without ascites (Fig. 1B, C). Contrast-enhanced computed tomography showed no evidence of biliary obstruction or structural abnormalities (Fig. 1D). The patient’s R value calculated from ALT and ALP levels was < 2, indicating a cholestatic pattern of liver injury. As his persistent hyperbilirubinemia could not be explained by biliary obstruction, liver biopsy was performed. Histological examination disclosed prominent canalicular cholestasis with mild portal inflammatory cell infiltration in the absence of bile duct destruction, interface hepatitis (Fig. 2A-C), and significant fibrosis (Fig. 2D, E). Based on the patient’s clinical history and laboratory findings, causality was assessed using the RECAM-J 2023 scoring system. Sildenafil received a score of 11 (Time to onset after drug initiation 4 points, Course after drug cessation 4 points, Previous evidence of hepatotoxicity 3 points, Exclusion of competing causes 0 points, Other supportive factors 0 points) and was classified as “highly probable,” in contrast to rosuvastatin and amlodipine, which yielded lower scores of 9 (Time to onset after drug initiation 2 points, Course after drug cessation 4 points, Previous evidence of hepatotoxicity 3 points, Exclusion of competing causes 0 points, Other supportive factors 0 points) and 7 (Time to onset after drug initiation 0 points, Course after drug cessation 4 points, Previous evidence of hepatotoxicity 3 points, Exclusion of competing causes 0 points, Other supportive factors 0 points), respectively. Rosuvastatin had been used continuously for two years without dose modification or previous liver test abnormalities, making it a less likely causative agent. Taken together, these findings supported a diagnosis of sildenafil-induced liver injury.

**Fig. 3:**
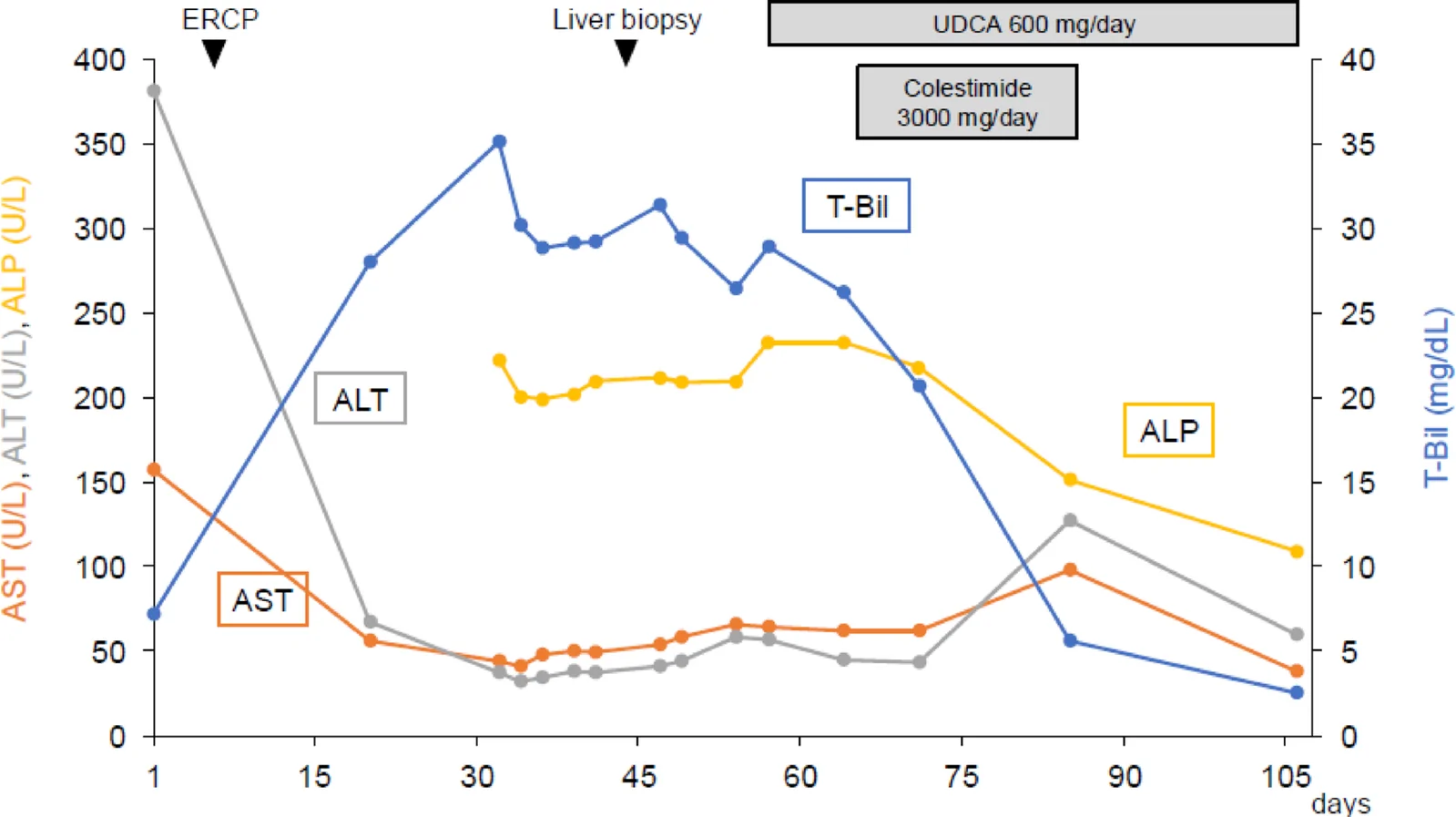
Clinical course. Changes in serum aspartate aminotransferase (AST, U/L; orange line), alanine aminotransferase (ALT, U/L; gray line), alkaline phosphatase (ALP, U/L; yellow line), and total bilirubin (T-Bil, mg/dL; blue line) levels are shown. The patient received ursodeoxycholic acid (UDCA, 600 mg/day) and colestimide (3,000 mg/day) as supportive therapy, after which these laboratory parameters gradually improved. Sildenafil had been discontinued before the patient's initial presentation (Day 1).

Ursodeoxycholic acid was initiated at 600 mg/day (Fig. 3). The patient’s liver enzymes and bilirubin gradually improved, with total bilirubin decreasing to 26.35 mg/dL before his discharge in stable condition on hospital day 10. Because cholestasis persisted two weeks after discharge, colestimide was added at 3000 mg/day. At the two-month follow-up, total bilirubin was 2.47 mg/dL, albumin was 4.1 g/dL, prothrombin time was 87.7%, and ALP was 109 U/L, all of which were indicative of continued improvement. His total bilirubin and ALP normalized approximately three months after symptom onset.

## Discussion

We report a rare case of severe cholestatic DILI associated with sildenafil obtained locally during the patient’s stay in the Philippines. The liver injury developed after choledocholithiasis-associated obstructive jaundice, making differentiation from prolonged obstructive cholestasis particularly challenging. The diagnosis was supported by the clinical course, RECAM-J 2023 assessment, and liver histology.

DILI is broadly classified as intrinsic or idiosyncratic, with the biochemical pattern of liver injury further categorized as hepatocellular, cholestatic, or mixed according to R value [10]. In the present case, marked hyperbilirubinemia with only mild elevations in transaminases yielded an R value of 0.5, indicating a cholestatic pattern consistent with the patient’s clinical and histological findings. Causality assessment using RECAM-J 2023 revealed a sildenafil score of 11, indicating a “highly probable” relationship with liver injury [11]. In contrast, rosuvastatin and amlodipine were considered less likely due to their long-term use without dose modification. Although a drug-induced lymphocyte stimulation test was negative, its limited sensitivity did not definitively exclude DILI, particularly in the case of cholestatic injury [12]. These findings highlight the importance of structured causality assessment based on clinical course, competing etiologies, and detailed drug exposure history. However, RECAM-J 2023 is a probability-based supportive assessment tool rather than a definitive diagnostic standard, and causality should ultimately be assessed based on the overall clinical context, including the clinical course and histopathological findings.

One particularly noteworthy feature of the present case was the diagnostic challenge posed by persistent jaundice following successful stone extraction. Despite resolution of the biliary obstruction, bilirubin levels continued to increase, and repeated imaging studies showed no evidence of recurrent obstruction. In addition, liver biopsy demonstrated predominant cholestatic changes without bile duct destruction, findings that favored cholestatic DILI over prolonged obstructive cholestasis. Interestingly, the patient exhibited marked hyperbilirubinemia despite only modest elevations in ALP and GGT, a pattern that may resemble bland cholestasis. The key differential features between cholestatic DILI and prolonged obstructive cholestasis are summarized in Table 2 [13–15].

**Table 2:** Cholestatic DILI versus prolonged extrahepatic obstruction

	Cholestatic DILI	Prolonged extrahepatic obstruction
Onset pattern [13]	Typically 30–90 days after drug initiation	Depends on the underlying cause
Response to drug withdrawal [14]	Improvement	No improvement until obstruction is relieved
AST / ALT [13]	Mild to moderate elevation	Often marked elevation, particularly in the early phase
Total bilirubin [15]	Variable elevation	Persistent and marked elevation
Histologic cholestasis (%) [13]	42	83
Imaging findings	No biliary obstruction on imaging	Dilated bile ducts and obstructive lesions

ALT, Alanine aminotransferase: AST, Aspartate aminotransferase: DILI, Drug-induced liver injury

Sildenafil is primarily metabolized in the liver via cytochrome P450 enzymes, mainly CYP3A4, and is generally well tolerated [16, 17]. Indeed, studies investigating a possible link between sildenafil use and liver damage are limited, and the underlying mechanism responsible for hepatotoxicity remains unclear [2]. We performed a literature search on PubMed using the terms (“sildenafil” OR “KAMAGRA”) AND (“drug-induced liver injury” OR “case report”), which uncovered several related cases [3–8]. The reports suggested that although uncommon, sildenafil-related liver injury could present with a cholestatic pattern, prolonged hyperbilirubinemia, and delayed recovery. Symptoms developed approximately one month after sildenafil initiation, consistent with the latency reported in previous cases. These features were also observed in the present case.

Another important clinical implication is the difficulty in obtaining an accurate history of erectile dysfunction medication use. Sildenafil-containing agents, over-the-counter sexual enhancement products, and medications obtained abroad are often underreported because of embarrassment, privacy concerns, or the perception that they are not conventional medications. Therefore, unless clinicians specifically inquire about such products, exposure may remain unrecognized during the evaluation of unexplained liver injury.

In addition, transient biliary obstruction and cholestasis may have contributed to increased systemic exposure to sildenafil in the present case. Sildenafil is primarily metabolized hepatically via CYP3A4, and impaired hepatic clearance under cholestatic conditions could theoretically elevate circulating drug concentrations and enhance susceptibility to hepatotoxicity. Although this mechanism cannot be proven in the current case, it may have played a contributory role in the development and severity of liver injury.

Several limitations should be considered when interpreting the present case. Although the patient had metabolic risk factors, including obesity (BMI 31.1 kg/m^2^), dyslipidemia, and hypertension, hepatic steatosis was not observed on liver biopsy. However, the absence of steatosis in a single biopsy specimen does not necessarily exclude underlying or prior MASLD, because hepatic fat deposition may be heterogeneously distributed and can fluctuate over time. In addition, sampling variability inherent to liver biopsy may have partially contributed to this discrepancy. Furthermore, although UDCA and colestimide were administered during the clinical course, these agents were used as empirical supportive therapies, and the primary management was withdrawal of the suspected causative drug.

In conclusion, sildenafil and similar products obtained outside regulated medical settings can cause severe and prolonged cholestatic DILI, potentially mimicking obstructive jaundice. Awareness of this potential adverse effect, careful causality assessment using structured scoring systems such as RECAM-J 2023, and thorough medication history taking are essential for accurate diagnosis and appropriate management.
